# Endemicity of *Taenia solium* cysticercosis in pigs from Mbeya Rural and Mbozi districts, Tanzania

**DOI:** 10.1186/s12917-020-02543-9

**Published:** 2020-09-03

**Authors:** Mwemezi L. Kabululu, Helena A. Ngowi, James E. D. Mlangwa, Ernatus M. Mkupasi, Uffe C. Braae, Chiara Trevisan, Angela Colston, Claudia Cordel, Maria V. Johansen

**Affiliations:** 1 Tanzania Livestock Research Institute (TALIRI) – Uyole, Mbeya, Tanzania; 2 Department of Veterinary Medicine and Public Health, College of Veterinary Medicine and Biomedical Sciences, Sokoine University of Agriculture, Morogoro, Tanzania; 3 Department of Infectious Disease Epidemiology, Statens Serum Institut, Copenhagen, Denmark; 4 One Health Center for Zoonoses and Tropical Veterinary Medicine, Ross University School of Veterinary Medicine, Basseterre, Saint Kitts and Nevis; 5 Department of Biomedical Sciences, Institute of Tropical Medicine, Antwerp, Belgium; 6 Global Alliance for Livestock Veterinary Medicines (GALVmed), Nairobi, Kenya; 7 Global Alliance for Livestock Veterinary Medicines (GALVmed), Bloemfontein, Free State South Africa; 8 Department of Veterinary and Animal Sciences, Faculty of Health and Medical Sciences, University of Copenhagen, Frederiksberg, Denmark

**Keywords:** *Taenia solium*, Cysticercosis, Pigs, Mbeya, Mbozi, Tanzania

## Abstract

**Background:**

*Taenia solium* taeniasis/cysticercosis is a disease of substantial economic and public health importance particularly in low–income countries. The disease was reported to be endemic in Mbeya Rural and Mbozi districts, in the southern highlands of Tanzania, the major pig production area in the country. In 2008, using B158/B60 antigen detection enzyme–linked immunosorbent assay (Ag–ELISA), porcine cysticercosis prevalence of up to 32% was reported in the districts. A number of interventions have been implemented in selected villages including an integrated approach consisting of improving pig confinement and selective treatment of pigs with oxfendazole. Mass drug administration with praziquantel targeting schistosomiasis, with an expected effect on *T. solium*, was also provided to school–age children in the area. This study aimed at providing an update on prevalence and intensities of porcine cysticercosis; and assessing farmers’ knowledge, attitudes and practices which could be associated to disease transmission in the area. The study involved a questionnaire survey conducted using face–to–face household interviews with 890 consenting farmers; and carcass dissections performed on 282 pigs randomly selected from the surveyed households.

**Results:**

Twenty–six pigs (9.2%) were infected with *T. solium*; of which two–thirds (65.4%) had light to moderate infection intensities (1–1000 cysticerci), and one–third (34.6%) had heavy intensities (> 1000 cysticerci). Questionnaire results showed that only 5.7% of the respondents perceived *T. solium* cysticercosis to be an important disease in pigs. About 18.5% of the respondents were aware of *T. solium* taeniasis, but 32% of them were unaware of how the infection is acquired. Half of the respondents had seen cysticerci in pork, of whom 61% were not aware that consumption of infected pork could cause taeniasis. Latrines were observed to often (90%) lack doors with 45% considered accessible to pigs.

**Conclusions:**

This study provided an evidence that the disease was still endemic in the area. Poor knowledge of farmers, attitudes, and risky practices responsible for disease perpetuation were also revealed. A One Health approach targeting the whole area incorporating improvement of farmer knowledge regarding disease transmission needs to be trialed as a feasible approach to control.

## Background

Taeniasis and cysticercosis caused by a zoonotic tapeworm *Taenia solium* are diseases responsible for major public health and economic burdens in endemic countries, including Tanzania. The life cycle of the parasite involves pigs and humans as intermediate and final hosts respectively, causing porcine cysticercosis (PC) in pigs and taeniasis in humans. Transmission occurs when pigs have direct or indirect access to human stool containing *T. solium* eggs and humans consume raw or poorly cooked pork infected with cysticerci. Accidental ingestion of the *T. solium* eggs by humans leads to human cysticercosis and when cysticerci lodge into the brain they cause neurocysticercosis (NCC), a potentially fatal form of the disease. *T. solium* taeniasis and cysticercosis are endemic in many countries of sub–Saharan Africa (SSA) [1], Asia [2] and Latin America [3].

In Tanzania, the disease in pigs was first reported in 1995 in the northern part of the country [4, 5]. The first survey in Mbeya Rural and Mbozi districts, in the southern highlands was conducted in 2007/2008 and reported a PC prevalence in pigs of up to about 11.7% based on lingual examination, and 32% based on B158/B60 antigen detecting enzyme–linked immunosorbent assay (Ag–ELISA) [6]. A further survey in the two districts in 2010/2011 reported a PC sero–prevalence of 25.5% [7].

In humans, prevalence of taeniasis of up to 5.2% based on copro–antigen ELISA, and cysticercosis prevalence of up to 16% based on Ag–ELISA or IgG Western Blot have been reported [8]. The major risk factors for *T. solium* taeniasis*/*cysticercosis in the area have been reported to be poor general knowledge on the disease and its transmission, free roaming pigs, lack/poor use of latrines, poor hygiene and use of unsafe water sources [6, 8, 9].

Due to its public health importance, *T. solium* was ranked highest on the global scale of food–borne parasites and was identified as a major cause of deaths from food–borne diseases [10, 11]. In SSA, it is estimated that about 6.2 million people suffer from NCC and about 30% of epileptic cases are due to NCC [12, 13]. Trevisan et al. [14] estimated that in Tanzania, approximately 5 million USD (95% Uncertainty Interval [UI], 797,535–16,933,477) were spent in 2012 due to NCC–associated epilepsy and additionally 3 million USD (95% UI, 1095, 960–5,366,038) were potentially lost due to PC.

Worldwide, there is an increased advocacy for controlling the disease. In Tanzania, *T. solium* taeniasis/cysticercosis was included on a list of the country’s research priorities for a period of 2015 to 2020 [15]. A number of intervention strategies have been trialed and implemented with varied short–term effects. An integrated approach involving improved pig housing (confinement), improved feeding and selective/strategic treatment of pigs with oxfendazole did not significantly reduce prevalence of PC when assessed seven and 14 months post–intervention [16]. In addition, mass drug administration (MDA) with praziquantel (at 40 mg/kg) to specifically target schistosomiasis has been provided to school children in the area since 2012, biennially in Mbeya Rural district and annually in Mbozi district. Praziquantel is also efficacious against *T. solium*, even at a lower dose of 5–10 mg/kg [17]. Assessment of the MDA suggested that three rounds of annual MDA could reduce prevalence of *T. solium* in pigs and humans hence a potential cost–effective option for control [18]. Sensitization of the farmers about the parasite’s transmission and importance has been provided to the community in the area since 2006.

This study aimed at estimating the current prevalence and infection intensities of PC in pigs in the two districts of Mbeya Rural and Mbozi. Further, the study sought to assess pig farmers’ knowledge, attitudes and practices, which may influence transmission and perpetuation of the disease in the area.

## Results

### General description of pig management practices

The 890 surveyed households/pig farmers had a total of 3094 pigs (supplementary file 1). Most of the pigs (80.7%) were crossbreeds of indigenous breeds and exotic pigs mainly Landrace and Large white. The majority (80.4%) of the respondents kept one to five pigs with a median of two pigs and a range of one to 37 pigs. Maize bran was the most common feed for pigs (mentioned by 83.9% of respondents), followed by food waste/kitchen leftovers (mentioned by 47.5% of respondents). In some cases, the maize bran was mixed with protein/mineral supplements, most commonly sunflower seed cake. Commonly, farmers used either maize bran or food waste, depending on availability. Rivers and wells were the main sources of drinking water for pigs as they contributed about 60% of water provided to pigs. Other sources included boreholes, rainwater, and communal piped water outlets.

Most (98.4%) farmers kept pigs for the purpose of selling them to cater for their financial needs. Weaner/grower pigs were usually sold to other farmers for breeding or finishing purposes. In most cases (99.7%), farmers sourced replacement pigs from within their villages. Slaughter pigs were being sold to local butchers; or to pig traders who then transported the pigs to urban areas including the country’s major city, Dar es Salaam, about 900 km from the study areas.

### Farmers’ knowledge, attitudes, and practices related to *T. solium* transmission

At the time of the visit, 59.2% (524/885) of the households confined their pigs in pens, 19.7% let their pigs to roam and 9.8% tethered their pigs. The rest practiced different combinations of the above including penning or tethering pigs for part of the day. According to our observations, confining pigs in pens was more practiced in Mbeya Rural district (81.8%, 296/362) than in Mbozi district (43.6%, 228/523) which also meant that free range pig management was more practiced in Mbozi district (26.2%, 137/523) than in Mbeya Rural district (10.5%, 38/362). The differences were statistically significant (χ^2^ = 99.2 and 45.5 respectively; *p* = 0.000 for both).

Overall, 5.7% (50/878) of the respondents mentioned *T. solium* cysticercosis to be among important diseases affecting their pigs. More respondents in Mbeya Rural district (9.5%, 34/359) than in Mbozi district (3.1%, 16/519) mentioned cysticercosis as an important pig disease (χ^2^ = 16.1, *p* = 0.002). About 18.5% (163/880) of respondents were aware of the taeniasis infection in humans, out of whom about one–third (32%) did not know how the infection was acquired. About half (53.4%) of the respondents who knew taeniasis associated it with consumption of raw/undercooked pork. The remainder mentioned other sources of infection such as drinking unclean water/raw milk and eating raw/undercooked beef and unwashed vegetables/fruits. A significantly higher proportion of respondents in Mbozi district (24.1%, 125/518) were aware of the tapeworm infection in humans than in Mbeya Rural district (10.5%, 38/362) (χ^2^ = 26.2, *p* = 0.003).

About half of the respondents (48.9%, 430/879) had seen cysticerci in pork; of whom 61.2% were not aware that consumption of the meat could cause disease in people. More respondents in Mbozi district (61%, 317/520) had seen cysticerci–infected pork as compared to respondents in Mbeya Rural district (31.5%, 113/359) (χ^2^ = 73.8, *p* = 0.000). About 58% of the respondents knew people in their localities who had epilepsy and/or other clinical manifestations associated with NCC and the proportion was higher in Mbozi district (73.4%) than in Mbeya Rural district (36.3%) (χ^2^ = 120.1, *p* = 0.000).

Most (86.7%, 743/857) respondents reported that they and their families consumed pork. However, home slaughter of pigs was rare, practiced only by 2% of the respondents. The majority (98%) of the respondents mentioned that they sourced pork from local butcheries/pork outlets situated around community gathering areas where villagers also bought other household needs. In most cases, pork was prepared and consumed on the spot in and around local bars. In a few instances, prepared pork was bought and taken home to be consumed by other family members. Sometimes, unprepared pork was bought to be prepared and consumed at home. Frying was the commonest method of preparing pork mentioned by 43.7% (376/860) of respondents, followed by a combination of boiling and frying (38.6%, 332/860) in which case pork was boiled first before being fried.

Most pig keeping households (95.4%, 840/881) had latrines and the respondents mentioned that the latrines were always (99.1%) used by all household members. More households in Mbozi district (99%, 516/521) had latrines compared to households in Mbeya Rural district (90%, 324/360) (χ^2^ = 39.2, *p* = 0.001). However, it was observed that most latrines (> 90%) had no doors or had doors which were left open when the latrines were not in use. As a result, it was determined that nearly half (45%) of the latrines in both districts could be accessible to pigs. Pig access to latrines was observed to be more probable in Mbozi district (61.7%, 317/514) than in Mbeya Rural district (19.2%, 63/329) (χ^2^ = 146.5, *p* = 0.000). Also, it was observed that only a few households had hand washing facilities by their latrines suggesting that washing hands after latrine use was not a common practice. Further, majority of the respondents (78.5%) did not wash their hands before feeding the pigs. Rather, more farmers (77.4%) washed their hands after feeding pigs.

### Pig necropsy

In total, 282 pigs were slaughtered and their carcasses dissected and examined. *T. solium* cysticerci were found in 26 pigs (9.2, 95% Confidence Interval [CI]: 6.1, 13.2). Mbozi district had higher prevalence (22/154, 14.3, 95% CI: 9.2, 20.8) than Mbeya Rural district (4/128, 3.1, 95% CI: 0.9, 7.8). The difference in prevalence between the two districts was statistically significant (*p* = 0.001, z = 3.23). Total number of cysticerci and distribution in the organs/muscle groups are shown in Table 1. Out of the 26 pigs with *T. solium* cysticerci, two had both viable and non–viable cysticerci and the rest had viable cysticerci only. Non–viable cysticerci accounted for less than 1 % of all cysticerci. Total numbers of cysticerci ranged from two to 38,730. About two–thirds (65.4%) of the infected pigs had light to moderate infection intensities and one–third (34.6%) had heavy intensities.

**Table 1:** Numbers of *Taenia solium* cysticerci in 26 infected pigs out of 282 slaughtered pigs in Mbeya Rural and Mbozi districts, Tanzania

Animal number	Total count of cysticerci	% viable cysticerci	Brain	Tongue	Masticatory muscles	Heart	Diaphragm	Forelimb	Remaining half carcass
5	116	100	1	1	6	10	10	36	52
19	2	100	0	0	0	0	0	2	0
21	97	94.8	2	1	2	5	3	26	58
25	10	80	0	1	0	1	0	0	8
31	12	100	1	0	0	0	1	2	8
44	2935	100	8	57	41	141	42	576	2070
130	2	100	0	0	0	0	0	0	2
149	3228	100	12	329	369	69	515	430	1504
150	19,603	100	148	1846	851	20	0	1980	14,758
157	4322	100	1	167	245	138	113	1174	2484
162	437	100	2	16	62	35	2	222	98
169	2162	100	17	94	112	48	17	1076	798
170	6	100	0	0	1	3	0	0	2
184	42	100	0	4	0	8	0	18	12
188	84	100	3	3	6	1	3	32	36
192	1114	100	2	96	143	167	2	430	274
194	3113	100	9	201	118	346	213	810	1416
195	246	100	0	13	14	8	5	94	112
200	26	100	0	0	0	0	0	6	20
202	4	100	0	0	0	0	0	0	4
210	5145	100	24	241	247	366	313	1968	1986
218	6	100	0	0	0	6	0	0	0
222	38,730	100	433	2750	6800	336	635	17,928	9848
224	110	97.3	0	8	3	3	2	34	60
226	4	100	0	0	0	4	0	0	0
236	2	100	0	2	0	0	0	0	0

Relative distribution, mean numbers and maximum counts of *T. solium* cysticerci in different organs and muscle groups among infected pigs are shown in Table 2. Apart from the musculature of the forelimb and the remaining half carcass, the heart was found to be the most frequently infected organ (76.9% of infected pigs) followed by the tongue. The brain was the least frequently infected organ, as cysticerci in brain were found in 54% of the infected pigs.

**Table 2:** Relative distribution, mean number and maximum counts of cysticerci in organs and muscle groups of 26 pigs infected with *Taenia solium* cysticerci in Mbeya Rural and Mbozi districts, Tanzania

Organ/muscle group	Relative distribution of cysticerci		Mean number of cysticerci	Std. Dev.^b^	Max.^c^
	Number^a^	%			
Brain	14	53.8	25.5	88.0	433
Tongue	18	69.2	224.2	629.9	2750
Masticatory muscles	16	61.5	349.9	1328.8	6800
Heart	20	76.9	66.0	114.8	366
Diaphragm	15	57.7	72.2	166.3	635
Forelimb	19	73.1	1032.5	3495.4	17,928
Remaining half carcass	22	84.6	1369.6	3378.9	14,758

^a^Number of infected pigs

^b^Std. Dev–Standard Deviation

^c^Max.–Maximum recorded number of cysticerci per organ/muscle group

## Discussion

The results presented in this study show that *T. solium* cysticercosis of pigs was persistent in Mbeya Rural and Mbozi districts and that farmers’ knowledge about its importance and transmission was poor. This study also revealed specific attitudes and risk practices among farmers, which probably contributed to perpetuation of the disease in the area. Hence, PC still presents a public health threat in this important pig producing area in Tanzania. This calls for a long–term One Health approach addressing pigs and humans in the whole area.

To our knowledge, this is the first study in Tanzania reporting prevalence of *T. solium* cysticercosis in pigs based on carcass dissection, the most definitive measure of PC prevalence and intensity [19]*.* Only two previous studies in Tanzania used carcass dissection to determine *T. solium* cysticercosis in pigs but none of them aimed to determine prevalence. The studies examined relatively small sample sizes and pigs were pre–selected by tongue palpation. A study by Boa et al. [20] determined distribution and density of cysticerci in 24 finished naturally infected pigs while Mkupasi et al. [21] used 61 naturally infected pigs to evaluate efficacy and safety of ivermectin and oxfendazole against cysticercosis and other parasitoses. As tongue palpation is known to have low sensitivity and usually fails to identify light infections [22, 23], the pigs slaughtered in the two studies were not really representing pig populations in the respective areas.

Necropsy results presented in this study suggest that the intensity of infection was aggregated; such that the majority of the cysticerci were harbored by a minority of pigs. Only one–third of the infected pigs had heavy infection intensities. Among other factors, intensity of cysticerci in an infected pig is related to an infection dose and heavy intensities are usually associated with direct ingestion of proglottids released by a tapeworm carrier [24]. This happens in areas where pigs are freely roaming and people practice outdoor defecation, or where defaecation is practiced within the pig confinement area as it has been reported in Cameroon [25]. However, the observed cysticerci aggregation points to the role of transmission modes other than direct ingestion of human stool. It was previously reported in the study area that pig confinement did not prevent pigs from being infected with *T. solium* and that transmission might occur through contaminated feeds and water provided to pigs [6, 9, 26]. Such infections are likely to be in lower doses of cysticerci resulting to light/moderate infection intensities. These infections are of a particular public health importance. Studies have shown that light/moderate infections are likely to be missed by tongue palpation and routine meat inspection and may consequently enter the food chain [20, 25, 27]. Studies are therefore warranted to determine the extent to which such carcasses may not be detected at official slaughter slabs in the study area and enter the food chain. Because of the cost and logistical limitations of the full carcass dissection, selected (partial) tissue dissection may be useful, as suggested by Lightowlers et al. [19]. In the present study, 22 out of the 26 infected pigs (84.6%) could be detected by dissecting only the tongue, the heart, and either the masticatory muscles or the diaphragm. This level of sensitivity is comparable with that reported in the study by Lightowlers et al. [19] where 31 of 38 (81%) of infected pigs were identified by only dissecting the tongue, masticatory muscles and the heart.

The reported low level of farmers’ knowledge on the disease transmission and its importance, presumably contributes to the reported attitudes and risk practices resulting in disease endemicity. This can be explained by, among other things, the fact that no specific education program has been provided for control of *T. solium* cysticercosis in the area. As a result, knowledge of the communities regarding the disease importance and transmission remained poor. Proper knowledge of pig farmers and communities in general on *T. solium* taeniasis/cysticercosis has been found to influence proper practices and is therefore crucial for control of the disease complex [28–31]. Specific health education tools against *T. solium* might therefore be useful, as studies with professionals have shown that knowledge can persist for a prolonged period following a health education program [32, 33]. Although the overall knowledge on *T. solium* was low, more farmers in Mbeya Rural district regarded the disease to be important. This can be assumed to be one of the reasons why more farmers confined their pigs in Mbeya Rural district than in Mbozi district. However, improvement in knowledge may not necessarily translate to significant changes in behaviors and practices [28, 29]. In that respect, emphasis should be put on influencing behaviors and practices change through demonstrating the health benefits and economic gains which would be expected as a result of the change [34]. This should be an important component of any health education program.

This study is reporting high levels of latrine coverage and use in the area, but with persistent transmission of PC. Earlier studies in Tanzania associated absence of latrines with occurrence of *T. solium* infections [6, 9]. However, other studies showed no difference in prevalence between households having and those lacking latrines [6, 35, 36]. Apart from the fact that the reported high level of use of the latrines could not be proved, open latrines present a risk for transmission. Therefore, although it is important for the communities to have and use latrines, presence of open latrines is probably counterproductive, as it was also showed by Braae et al. [9]. This is more important in areas where pigs roam freely as the chances of pigs having access to the latrines are increased. In this study, more pigs could access the latrines in Mbozi district than in Mbeya Rural district, which points to the ease of which transmission occurs from human to pigs being different in the two districts. Hence, the communities should be educated on the importance of not only having latrines, but also proper use of the latrines, such as ensuring that latrines have properly closing doors, and latrine doors remain closed. In addition, local by–laws governing use of latrines and prohibiting pig free roaming should be formulated (where not in place) and enforced.

As it has been shown in this study, lack of hand washing before feeding pigs and more importantly after latrine use was common and is probably a disease risk factor. It is therefore important to emphasize washing hands with soap after latrine use, before eating and before feeding pigs. However, sources of water that were used in the communities might reduce the usefulness of washing hands with water as, water of such sources may carry disease pathogens such as *Taenia* eggs from the contaminated environment [37, 38]. The extent to which the environment in the study area is contaminated with *Taenia* eggs is not yet reported, but earlier studies have associated use of the water with higher sero–prevalence in pigs [6] and in humans [8]. The information on the role of the environment is needed to fully elucidate transmission dynamics of *T. solium* in the study area. More importantly, a One Health approach is needed for control of *T. solium*, whereby veterinary and medical professionals work together to address both hosts, pigs and humans; and the environment.

Limitations of this study included the fact that musculature of only half of the carcasses were dissected. Studies in Zambia and Nepal reported that half carcass dissection can miss 16 and 25% of positive cases, respectively [25, 39]. Therefore, it may be assumed that some of lightly infected cases might have been missed. In addition, as weights of different organs/muscle groups were not measured, information on the cyst density which would provide more accurate information on cyst predilection and distribution is lacking. Further, infected pigs could not be traced back to the level of their originating households hence differences in risk and protective factors between households with and without infected pigs could not be quantified.

## Conclusions

In conclusion, this study showed that *T. solium* cysticercosis in pigs was still prevalent in this important pig production area in Tanzania. This study also revealed that farmers‘knowledge on the disease was poor and this could be associated with the risky practices and attitudes which were observed. This calls for more interventions to safeguard health of the communities. Due to zoonotic nature of the *T. solium,* obtaining control and eventually elimination of the disease seems more feasible and sustainable through a One Health approach targeting both porcine and human hosts, and the environment.

## Methods

### Description of the study area

This study was conducted in Mbeya Rural and Mbozi districts of Mbeya and Songwe regions, respectively, in the southern highlands of Tanzania. Earlier studies have reported the disease to be endemic in the two districts [40]. Mbeya Rural district was comprised of 30 wards and 172 villages while Mbozi district consisted of 29 wards and 123 villages. In the 2012 census, the human population was recorded to be 305,319 in Mbeya Rural district and 446,339 in Mbozi district [41]. Both districts are largely rural with crop production and livestock keeping as the main economic activities. About 36.8 and 55.5% of households engage in livestock keeping in Mbeya Rural district and Mbozi district, respectively. Pig production is predominantly on a small scale and in 2016 pig population was estimated to be 16,935 in Mbeya Rural district and 26,930 in Mbozi district (Unofficial data, District Livestock Offices).

### Study design

This was a cross–sectional study involving a questionnaire survey and pig necropsies which were conducted between October and December 2016. The study was conducted as a baseline survey for an intervention trial which aimed at evaluating effectiveness of TSOL18 vaccination and oxfendazole treatment in pigs in reducing prevalence of *T. solium* cysticercosis of pigs in the two districts. Selection of villages was based on pig numbers, accessibility, previous studies and reports on the occurrence of *T. solium* cysticercosis in pigs. As much as it was possible, selection excluded villages which had been included in previous interventions [7, 16, 18]. Based on the mentioned inclusion and exclusion criteria, 16 villages, eight in each district, were selected for the study. In each village, pig farmers/pig keeping households were identified and listed with the help of village leaders and agricultural/livestock extension officers. All listed pig keeping households were visited and after the study objectives were explained pig farmers who were willing to participate were enrolled for the study. A total of 890 households were included in the study, and these constituted at least 90% of pig keeping households in the selected villages. In each household, a person who owned the pigs was interviewed, and in most cases this was the household head.

Slaughter pigs were purchased from households selected by using computer–generated random numbers from among the surveyed households. In each selected household, one eligible pig was bought for slaughter. An eligible pig was at least 6 months of age, non–pregnant and apparently healthy. In most cases a household had one pig that was eligible. In cases where there was more than one eligible pig, the farmer was requested to pick one pig he/she was willing to sell. A total of 282 pigs were purchased for slaughter.

### Household survey

Household data were collected by using a structured questionnaire (supplementary file 1), which was filled during door–to–door, face–to–face interviews. The questionnaire was in English but the interviews were conducted in Swahili (the national language) by trained extension officers, who were conversant in both English and Swahili. The questionnaire was pre–tested among enumerators first and then on pig keepers in a nearby village (not selected for the study) before it was administered to study respondents. Household particulars including family name and Global Positioning System (GPS) coordinates were recorded. Information gathered included pig management practices and farmers’ knowledge, attitudes and practices that might influence transmission of *T. solium* in the area. Observations were made to confirm interviewee responses particularly on pig confinement, presence of latrines; and to determine possible access of pigs to the latrines and presence of hand washing facilities adjacent to latrines.

### Pig slaughter and carcass dissection procedures

Slaughter pigs were bought from owners and were transported to a holding facility at Tanzania Livestock Research Institute (TALIRI), Uyole centre, Mbeya. They were then taken to a nearby public slaughter facility where they were slaughtered and processed as per the slaughter facility’s procedures. The heart and diaphragm were separated and transferred into labeled containers, and they were, together with the carcasses, transported to a post mortem facility at TALIRI–Uyole. At the facility, the tongue, brain, and masticatory muscles were extracted and labeled.

The carcasses were longitudinally partitioned into two halves. The muscles from the right half of the carcass were excised from bones, with muscles of the forelimb separated from muscles of the remaining half carcass. All muscles and inner organs were dissected with sagittal fine cuts of maximum 0.5 cm in thickness. The cut surfaces were macroscopically examined for presence, viability and number of *T. solium* cysticerci. A cysticercus was recorded as viable if it appeared translucent with a visible scolex in transparent fluid. A non–viable cysticercus appeared smaller, non–translucent, filled with dense whitish to yellowish fluid, or containing fibrous or caseous (calcified) material [42]. In case of an obvious heavy infection, for musculature in excess of one kg, a representative sample weighing one kg was sliced and cysticerci counted as above, and number of cysticerci for the remaining muscle mass was estimated based on its weight. Total cysticerci count for a pig was estimated as double the number for half carcass musculature plus numbers for the brain, tongue, masticatory muscles, diaphragm and the heart. For the purpose of this paper, a pig was considered infected if it had one or more viable cysticerci or more than one non–viable cysticercus in examined muscles/organs. Intensity of infection was categorized as light to moderate if total cysticerci count was between one and 1000 and heavy if it was more than 1000.

### Data analysis

Data was transcribed into Excel spreadsheets and imported into STATA© Version 12 for analysis. Descriptive analyses were used for determining frequencies and proportions, mean numbers of cysticerci (with standard deviations), maximum counts and ranges cysticerci numbers. Relative distribution of cysticerci for each organ or muscle group was determined as the number and proportion of infected pigs which had cysticerci in that organ/muscle group.

Prevalence results were compared between districts by using the two–group binomial test. Frequencies and proportion of questionnaire responses within the categories of questions and between districts were analysed by cross–tabulations and relevant associations were tested using a Chi–square (χ2) test for independence.

For all comparisons, a two–sided *p*–value < 0.05 was considered statistically significant and confidence intervals were computed at a 95% level of significance.

## Supplementary information

**Additional file 1.** A site questionnaire administered to selected pig farmers in the study area. The questionnaire had 29 questions divided into three sections namely; household particulars pertaining to porcine cysticercosis, pig management and care, and knowledge of taeniosis and cysticercosis.

## Data Availability

The datasets generated and analyzed during the current study are available from the corresponding author, Mwemezi Kabululu, E mail: mwemezie@gmail.com on a reasonable request.

## Abbreviations

PC: Porcine cysticercosis
NCC: Neurocysticercosis
SSA: Sub–Saharan Africa
Ag–ELISA: Antigen detecting enzyme–linked immunosorbent assay
UI: Uncertainty Interval
MDA: Mass Drug Administration
GPS: Global Positioning System
TALIRI: Tanzania Livestock Research Institute
CVMBS: College of Veterinary Medicine and Biomedical Sciences
DVS: Director of Veterinary Services
CI: Confidence Interval
