# Supplementary material for: Endemicity of Taenia solium cysticercosis in pigs from Mbeya Rural and Mbozi districts, Tanzania
Source: BMC Vet Res. 2020 Sep 3;16:325. doi: 10.1186/s12917-020-02543-9 (PMC7470673; doi:10.1186/s12917-020-02543-9)
Supplement: Supplementary file 1 — Additional file 1. A site questionnaire administered to selected pig farmers in the study area. The questionnaire had 29 questions divided into three sections namely; household particulars pertaining to porcine cysticercosis, pig management and care, and knowledge of taeniosis and cysticercosis. [file 12917_2020_2543_MOESM1_ESM.docx]

## ADDITIONAL FILE 1

**A site questionnaire administered to selected pig farmers in the study area**

**SECTION 1: HOUSEHOLD PARTICULARS PERTAINING TO PORCINE CYSTICERCOSIS**

1. GPS location

|  |
| --- |

1. Family name

|  |
| --- |

1. Does the household have a latrine?

Yes No

1. If used, is it used by household members

Always

Sometimes

Never

1. Do pigs have access to the latrine?

Yes No

**SECTION 2: PIG MANAGEMENT AND CARE**

|  |
| --- |

1. Give the current number of pigs in the household:
2. What breed of pig do you own?

|  | **YES** | **NO** |
| --- | --- | --- |
| Indigenous only |  |  |
| Exotic only |  |  |
| Crossbreed only (mix of indigenous and exotic by breeding) |  |  |

1. How do you keep your pigs?

|  | **YES** | **NO** |
| --- | --- | --- |
| Free ranging |  |  |
| Penned for part of day |  |  |
| Tethered for part of day |  |  |
| Intensive (zero foraging) |  |  |

1. Do you ever purchase replacement pigs?

Yes No

1. If yes, from where?

Purchase from within the neighbourhood

Purchase from outside the neighbourhood

If from outside neighbourhood please specify where:

|  |
| --- |

1. What is the reason/s for keeping the pig/s:

|  | **YES** | **NO** |
| --- | --- | --- |
| Home consumption |  |  |
| For sale |  |  |
| Breeding |  |  |

Other (specify)

|  |
| --- |

1. If pigs are slaughtered at home, is the meat inspected after slaughter before consumption?

Yes No

1. What feed types do you feed to your pigs:

|  | **YES** | **NO** |
| --- | --- | --- |
| Household leftovers or waste |  |  |
| Commercial feed |  |  |
| Swill (kitchen refuse and scraps of waste food mixed with water for feeding to pigs) |  |  |
| Scavenges |  |  |

1. Do you wash your hands before feeding the pigs?

Yes No

1. Do you wash your hands after feeding the pigs?

Yes No

1. Where does the pig’s water come from:

|  | **YES** | **NO** |
| --- | --- | --- |
| Provided from Household water |  |  |
| Municipal water supply point |  |  |
| Borehole |  |  |
| River |  |  |
| Rainwater |  |  |
| Wells |  |  |

Other (specify)

|  |
| --- |

1. Have you ever bought animal health products for your pigs?

Yes No

1. Do you use anthelminthics (substances capable of destroying or eliminating parasitic worms) in your pigs:

Yes No

1. Do you use anthelmintics (substances capable of destroying or eliminating parasitic worms) in people?

Yes No

1. What is the major disease affecting your pigs:

Cysticercosis

Malnutrition

Worms

Other/s (specify)

|  |
| --- |

1. Will you consider/be willing to pay for pig health products if you perceived this intervention to be successful?

Yes No

**SECTION 3: KNOWLEDGE OF TAENIOSIS AND CYSTICERCOSIS**

1. Do you know of tapeworm infestation/infection in humans?

Yes No

1. If yes, how is tapeworm infection acquired?

|  | **YES** | **NO** |
| --- | --- | --- |
| Eating raw or undercooked pork |  |  |
| Drinking raw milk |  |  |
| Eating raw or undercooked beef |  |  |
| Drinking unclean water |  |  |
| Eating unwashed vegetables and fruit |  |  |
| I do not know? |  |  |

Other (specify)

|  |
| --- |

1. Is pork consumed by you or members of your household?

Yes No

1. If yes, what is the source of the pork:

|  | **YES** | **NO** |
| --- | --- | --- |
| Home slaughter |  |  |
| Local butcheries |  |  |

Other (specify)

|  |
| --- |

1. If yes, how is it processed?

|  | **YES** | **NO** |
| --- | --- | --- |
| Boiled |  |  |
| Fried |  |  |
| Roasted |  |  |

Other (specify):

|  |
| --- |

1. Have you ever seen cysts (measles) in pig meat from your pigs?

Yes No

1. If yes, are you aware that if eaten, this meat can cause disease in people?

Yes No

1. Do you know of people complaining of epilepsy, chronic headaches, madness, seizures or skin nodules?

Yes No
